# The disjunctive cause criterion by VanderWeele: An easy solution to a complex problem?

**DOI:** 10.1007/s10654-019-00501-w

**Published:** 2019-03-05

**Authors:** Mohammad Arfan Ikram

**Affiliations:** Department of Epidemiology, Erasmus University Medical Center, PO Box 2040, 3000 CA Rotterdam, The Netherlands

**Keywords:** Confounding, Confounder selection, Causal inference, Etiology

Epidemiology is a discipline that is often as simple in its basic theory as it is complicated in its practical translation. Many practitioners of epidemiology, while entirely at ease with reproducing the theoretical constructs, find it difficult to translate these into practical steps for their research.

A case in point is the control for confounders in etiologic observational research. Confounding bias is a fundamental principle taught in any course or textbook of epidemiology, yet there is enormous variation in the applied literature in the way confounding is dealt with. In simple terms, a confounder is a common cause of the exposure and the outcome, and one of the solutions for confounding bias is to control for the appropriate confounder(s) as covariate(s) in regression models. In contrast to this simplicity of definition and possible solution, there is a plethora of methods on how to select an appropriate set of covariates that when controlled for in regression models sufficiently remove confounding bias from the associations under study. It is this antithesis between theory and practice which underlies the inconsistencies in the applied literature regarding control for confounding. I re-emphasize here that these inconsistencies are often not due to lack of theoretical knowledge about confounding, but rather due to lack of consensus in the field on practical application of said knowledge. In fact, overlooking my own research from the last decade, I can point towards some variation in the way covariates were selected [1–3].

In this issue of the *Eur J Epidemiology*, VanderWeele proposes a new approach for covariate selection in order to control for confounding [4]. Attempting to strike a balance between too liberal selection of covariates resulting in M-bias and too conservative selection resulting in insufficient control, he introduces the “disjunctive cause criterion” for selection of covariates. This criterion postulates that sufficient control for confounding can be achieved by “*controlling for each covariate that is a cause of the exposure, or of the outcome, or of both; excluding from this set any variable known to be an instrumental variable; and including as a covariate any proxy for an unmeasured variable that is a common cause of both the exposure and the outcome*”.

The theoretical framework underlying this criterion is elegantly explained in the original paper and this shows indeed that the disjunctive cause criterion selects a set of covariates that sufficiently controls for confounding. In the remainder of this commentary I will remark on several practical considerations, both strengths and challenges, that researches may encounter when applying this criterion, especially in biomedical research. These remarks are not intended to be exhaustive, but rather indicative.

With confounders being common causes between exposure and outcome, selection of confounders invariably requires knowledge of those causes as well as intermediates leading from those causes to exposure and outcome. In my opinion, the single most important advantage of the disjunctive cause criterion is that the amount of knowledge required for making the selection of covariates is much less compared to other criteria for covariate selection. With observational research entering an era of high-dimensional and multi-layered omics research the importance of this advantage cannot be downplayed. Biological systems—irrespective of whether considered at the level of a single cell, tissue, organ, or organism—appear to be ever so intricate and highly complex. Complete knowledge of the causal framework underlying the association under study, especially with respect to common causes, will essentially be impossible. The disjunctive cause criterion partly circumvents this problem by considering causes of the exposure and causes of the outcome separately, without the absolute necessity to have knowledge how these possibly different sets of causes could be linked to each other to result in *common* causes. From a practical point of view, this means that researchers might ascertain—from previous literature or any other source—the causes of the exposure separately from the causes of the outcome and still be reasonably reassured that they end up with a sufficient set of covariates to control for confounding, i.e. common causes. This is a much lighter task than requiring researchers to take a next step and synthesize existing knowledge to try to link those two sets of causes to identify common causes.

Note however, that even for the disjunctive cause criterion some knowledge of *common* causes is still required in order to meet the qualification “*include as a covariate any proxy for an unmeasured variable that is a common cause of both the exposure and the outcome*”. Nevertheless, controlling for non-common causes of the exposure or the outcome will likely account for many common causes already, which means that the amount of knowledge required to account for the remaining common causes will be much less as compared to situations where other criteria are used for covariate selection.

**Fig. 1:**
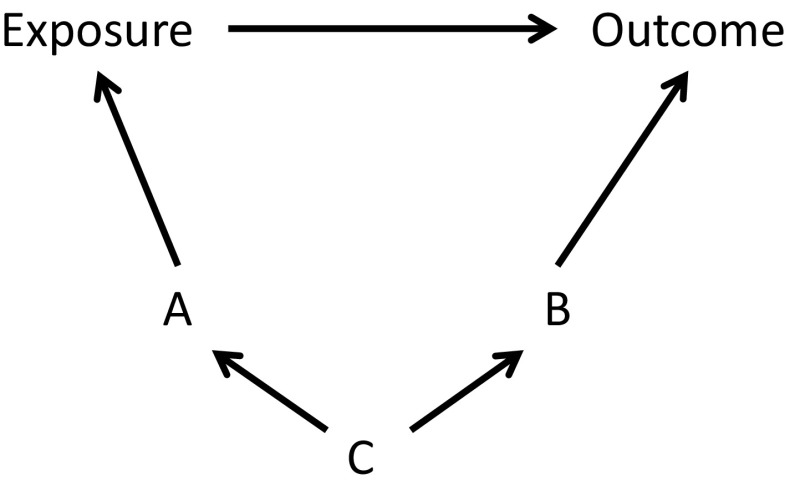
Causal diagram

There is also a possible challenge in approaching the causes of exposure and outcome separately. While the selection of covariates will be sufficient to adequately control for confounding, there is likely to be redundancy in this selection. For instance, applying the disjunctive cause criterion to the causal diagram in the Fig. 1 will identify covariates A and B to be controlled for. However, for adequate control for confounder C it is sufficient to control for only A, or only B (or of course only C). An underlying assumption here is that A nor B are affected by other confounders besides C. Practically speaking, too much redundancy in covariate selection might result in statistical challenges, such as collinearity and non-collapsibility. It is therefore wise for researchers to critically assess the list of covariates selected through the disjunctive cause criterion. If prior knowledge of the underlying causal framework unequivocally indicates redundancy in this selection, it might be worthwhile to adjust for only one among the redundant covariates.

A final consideration that I may highlight in the practical application of the disjunctive cause criterion and that was touched upon already by VanderWeele [4] is that most studies have a single concomitant measurement of the exposure and (possible) confounders. As pointed out by VanderWeele [4], in such instances discerning confounders from mediators can be a challenge. If the covariate in fact is a confounder, then adjusting will better provide the true effect size; conversely if the covariate actually is a mediator then adjusting may spuriously attenuate the true effect. In absence of any other possibility to discern a confounder from a mediator, i.e. data from prior waves, a pragmatic approach for researchers could be to report effect sizes both with and without adjustment of the covariate in question, while highlighting the causal framework that underlies the two models.

Notwithstanding the above remarks, the disjunctive cause criterion has some very strong properties regarding appropriate selection of covariates. I have no doubt that it will find widespread application in the field of biomedical research and beyond. Yet, the biggest contribution of the disjunctive cause criterion may turn out not to be the long sought-after consensus regarding covariate selection. Instead, the main contribution is perhaps that here is an example in epidemiology that is as easy to understand theoretically as it is to apply practically.
